# Influence of Solidification Conditions on the Microstructure of Laser-Surface-Melted Ductile Cast Iron

**DOI:** 10.3390/ma13051174

**Published:** 2020-03-06

**Authors:** Damian Janicki, Jacek Górka, Waldemar Kwaśny, Wojciech Pakieła, Krzysztof Matus

**Affiliations:** 1 Department of Welding, Silesian University of Technology, Konarskiego 18A, 44-100 Gliwice; Poland; jacek.gorka@polsl.pl; 2 Department of Engineering Materials and Biomaterials, Silesian University of Technology, Konarskiego 18A, 44-100 Gliwice, Poland; waldemar.kwasny@polsl.pl (W.K.); wojciech.pakiela@polsl.pl (W.P.); krzysztof.matus@polsl.pl (K.M.)

**Keywords:** laser surface melting, ductile cast iron: secondary cementite, tertiary cementite, thermography, cooling rate

## Abstract

The thermal conditions in the molten pool during the laser surface melting of ductile cast iron EN-GJS-700-2 were estimated by using infrared thermography and thermocouple measurements. The thermal data were then correlated with the microstructure of the melted zone. Additionally, the thermodynamic calculations of a Fe-C-Si alloy system were performed to predict the solidification path of the melted zone. It was found that increasing the cooling rate during solidification of the refined ledeburite eutectic but also suppressed the martensitic transformation. A continuous network of plate-like secondary cementite precipitates and nanometric spherical precipitates of tertiary cementite were observed in regions of primary and eutectic austenite. The solidification of the melted zone terminated with the Liquid → γ-Fe + Fe_3_C + Fe_8_Si_2_C reaction. The hardness of the melted zone was affected by both the fraction of the retained austenite and the morphology of the ledeburite eutectic.

## 1. Introduction

Over the past three decades, considerable research has been devoted to enhancing the wear resistance of different grades of cast iron via laser surface modification methods. Most reported works have focused on shaping the microstructure, and thus wear properties, of surface layers by using laser surface melting (LSM) [1,2,3,4,5]. This process involves rapid solidification and cooling, and it provides a unique opportunity to synthesize non-equilibrium structures [6,7]. Additionally, compared with other laser surface modification methods, such as laser surface alloying and cladding processes, LSM is a relatively simple process since it does not change the chemical composition of the processed surface layer [8,9,10]. However, the inability to change the chemical composition of the melt also significantly limits the ability to change the microstructure and mechanical properties of the processed surface layer [11]. During LSM, the mechanical properties of the processed layers can typically only be modified by controlling the solidification conditions in the molten pool [12,13]. Consequently, to optimize the wear properties of the working surface of the components that are made of a particular cast iron grade via LSM, it is necessary to comprehensively analyze the microstructure evolution in the processed surface layers under different solidification conditions. Unfortunately, especially in the case of ductile cast iron (DCI), little data are available. Since DCI is finding use in a growing range of industrial applications, comprehensive knowledge of the phenomena that occur during this process would be particularly useful. Such knowledge would provide a means to determine how to shape the microstructure, and thus the mechanical properties, of the resulting surface layers.

The thermal conditions in the molten pool of a DCI sample were analyzed by using infrared (IR) thermography. Since emissivity is the most important calibration parameter for temperature measurements when using IR thermography [14,15], this research method included an original calibration procedure of the IR camera to estimate the emissivity values of the melt in the molten pool, as well as regions that were directly adjacent to the molten pool under a given shielding atmosphere.

The main purpose of this work was to correlate changes in the selected processing conditions and the resulting changes in the solidification conditions in the molten pool with the microstructure of single-pass melted beads (SMB). To comprehensively understand the microstructure development in the melted zone, the developed research methodology combined an experimental approach with computational thermodynamics.

## 2. Materials and Methods 

For the research, DCI-grade EN-GJS-700-2 with a pearlitic/ferritic matrix and graphite precipitates with an average diameter of about 30 µm was selected. The chemical composition of the studied DCI substrate is given in Table 1. Substrate material (SM) specimens with dimensions of 25 × 60 × 10 mm were ground to an average roughness (*R*_a_) of approx. 0.5 µm. Prior to laser processing, the SM specimens were cleaned in acetone.

The laser processing trials were carried out by using an experimental stand that was equipped with a Rofin-Sinar DL 020 2.0 kW continuous-wave high-power direct diode laser (HPDDL, Rofin-Sinar Laser GmbH, Hamburg, Germany). The HPDDL had a rectangular beam with a top-hat intensity distribution in the slow-axis direction and a near Gaussian distribution in the fast-axis direction (spot size of 1.5 × 6.6 mm). Additional laser source details are available elsewhere [16]. During all trials, the slow-axis of the laser beam was set to be perpendicular to the traverse direction, and the beam focal plane was located at the SM specimen surface. Argon was used to prevent the laser-processed area from oxidation. The processing conditions are presented in Table 2. 

In order to examine the thermal conditions in the molten pool, the experimental stand was equipped with an original measurement system that combined IR thermography and a thermocouple. The system used an IR FLIR A600-Series camera (FLIR System, Inc. Wilsonville, OR, USA) with a measurement temperature range from 600 to 2200 °C and a spectral range of 7.5–14.0 µm. The maximum frame rate of the thermal image capturing system was 200 Hz. The thermocouple data were acquired and recorded by using a computer-based system with an Agilent 34970A data logger (Agilent Technologies, Inc., Santa Clara, CA, USA), which provided a maximum sampling frequency of 300 Hz.

**Figure 1:**
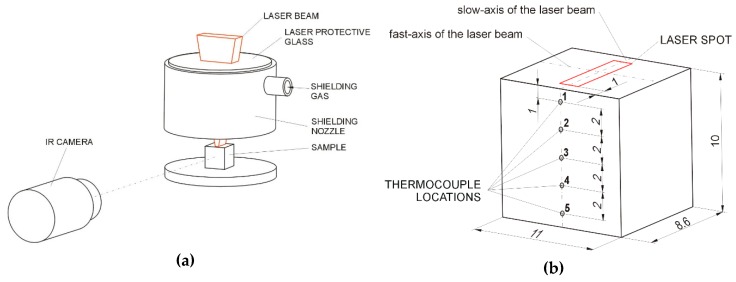
Diagram showing (**a**) the experimental setup for emissivity measurements of the ductile cast iron (DCI) in the as-received condition and after laser processing (during IR camera measurements) and (**b**) the sample dimensions, thermocouple locations, and alignment of the sample relative to the laser beam spot.

An FLIR A600-Series thermal camera was used to investigate the temperature on the surface of the molten pool and the regions that were directly adjacent to the molten pool on the laser-processed surface. To calibrate the IR camera, an original calibration procedure was developed that allowed for the estimation of the emissivity of the melt in the molten pool and the regions that were directly adjacent to the molten pool under a given shielding atmosphere. The emissivity data were used to reconstruct two-dimensional temperature profiles along the molten pool and the surrounding regions on the laser-processed surface. The developed procedure provided a direct correlation between the data of the IR camera and the thermocouple measurements. The scheme of this method is presented in Figure 1. During the above calibration procedure, the HPDDL beam heated and partially melted the sample under an argon atmosphere that was provided by the shielding nozzle that was arranged coaxially with the laser beam (Figure 1a). The dimensions of the sample and its alignment relative to the laser beam spot are presented in Figure 1b. By using the samples of the as-received DCI and the samples with the melted layer, it was possible to estimate the emissivity of the region in front of the molten pool and the solidified material of the processed bead behind the molten pool. The two-step calibration procedure included IR thermography and thermocouple measurements that required the use of samples with identical dimensions. In the first stage, an IR camera recorded the temperature fields during the heating and melting of a sample. In the second stage of the procedure, by using the same heating conditions of the sample, the thermocouple provided a pointwise check of the temperature fields that were previously recorded by the IR camera. The data from these two measurement stages were acquired and recorded at a rate of 200 Hz. The acquisition and recording of the thermocouple data were performed by using a computer-based system with an Agilent 34970A data logger. Depending on the location of the measuring point on the sample (Figure 1b) and the resulting range of recorded temperatures, a standard K-type thermocouple and non-standard high-temperature Mo–W thermocouple were used. The Mo–W thermocouple was calibrated by using the data reported by Michalski et al. [17].

Microstructural characterization was performed by using scanning electron microscopy (SEM) and transmission electron microscopy (TEM). SEM investigations were conducted by using a Phenom ProX (Phenom-World, Eindhoven, Netherlands) and ZEISS SUPRA 35 (Carl Zeiss Microscopy GmbH, Jena, Germany) microscopes. SEM observations were carried out in the backscattered electron (BSE) and in-lens image modes. For the SEM investigation, samples were polished by using 0.04 μm colloidal silica and etched with 12% Nital. An area (volume) fraction of the eutectic cementite phases was calculated from the SEM micrographs by using the NIS-Elements image processing software (version 3.13, Nikon Corporation, Tokyo, Japan).Measurements were performed on the SEM images of the undersurface area of the SMBs (~200 µm beneath the bead surface) over a total area of 10 mm² for each bead. The TEM analysis was conducted by using an FEI Titan 80/300 microscope (Scientific and Technical Instruments, Hillsboro, OR, USA). During the TEM investigation, the selected area electron diffraction (SAED) patterns were recorded for phase identification. The phase composition of SMBs and the unit cell parameters of the austenite phase were determined by using X-ray diffraction (XRD). The XRD analysis was performed using a PANalytical X’Pert PRO MPD X-ray diffractometer (Malvern PANalytical, Malvern, UK). XRD patterns were recorded by using an X’Celerator detector (Malvern PANalytical, Malvern, UK) and a Cu-K*_α_* (λ = 0.15406 nm) source operating at 40 kV and 30 mA. The XRD data were obtained over an angular range of 30–125°. The martensite and retained austenite fractions in the SMBs were evaluated by using the Rietveld refinement method in the FullProf software. 

Hardness measurements were conducted on polished SMB cross-sections by using a Wilson Wolpert 401 MVD Vickers microhardness indenter (Wilson Wolpert Instruments, Aachen, Germany) with a 200 g load and a 10 s dwell time.

Thermodynamics calculations of the Fe-C-Si ternary alloy system were made under both metastable equilibrium (considering the formation of the cementite phase) and non-equilibrium conditions by using commercial Thermo-Calc software. The Scheil–Gulliver model was used to predict the non-equilibrium solidification path of the Fe-C-Si system and to estimate the melt composition in the final stage of solidification. 

## 3. Results and Discussion

### 3.1. Examination of Thermal Conditions in the Molten Pool

**Figure 2:**
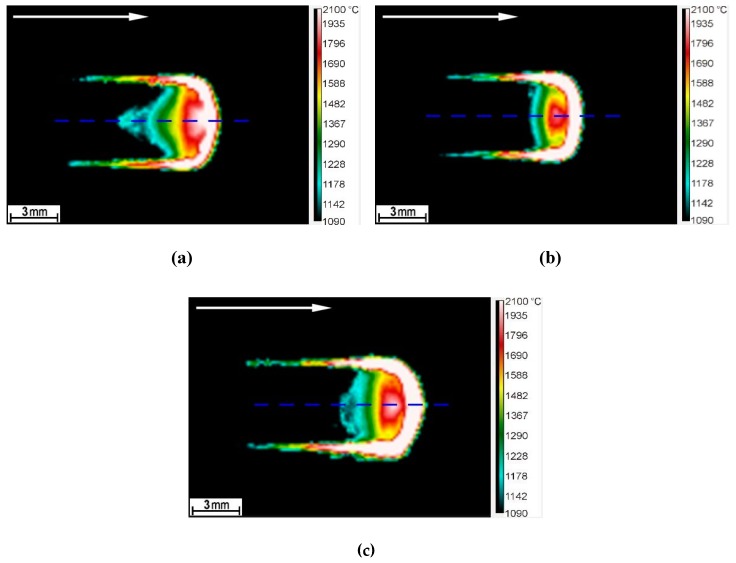
IR camera images obtained during LSM of the DCI under processing conditions: (Table 2) (**a**) A1, (**b**) A2, and (**c**) A3. (Selected emissivity value, *E* = 0.13). The arrows and dashed lines indicate the traverse direction of laser beam and the single-pass melted beads (SMB) centerline, respectively.

**Figure 3:**
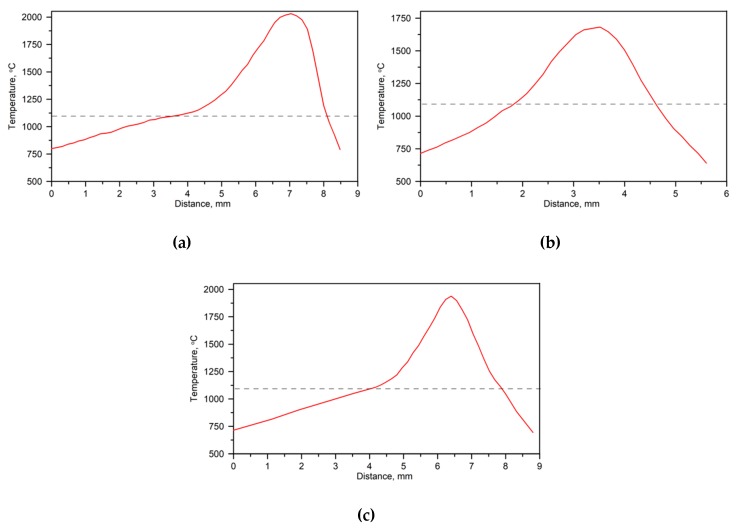
Temperature profiles at the surface of the molten pool along the SMB centerline under processing conditions: (Table 2) (**a**) A1, (**b**) A2, and (**c**) A3. Emissivity values were selected based on Figure 4. The dashed lines indicate the solidus temperature of the DCI that was determined by using the Scheil calculation (Section 3.2).

IR thermography measurements of the temperature distribution on the surface of the molten pool during LSM under processing conditions A1, A2, and A3 (Table 2) are presented in Figure 2. The corresponding temperature profiles at the surface of the molten pool along the SMB centerline are shown in Figure 3. 

**Figure 4:**
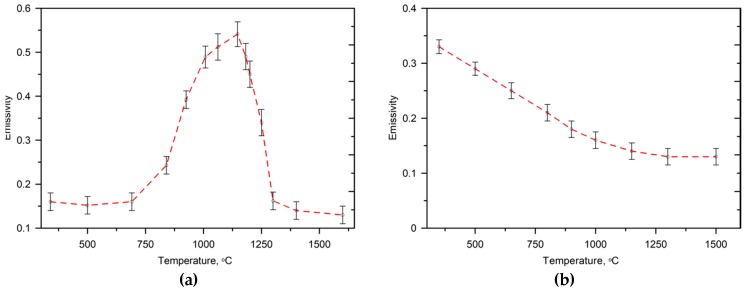
Variation of the emissivity of the surface of the investigated DCI as a function of temperature in (**a**) the as-received condition (after grinding to a surface finish of 0.5 µm *R*_a_) and (**b**) after laser surface melting.

Figure 4 presents the estimated emissivity values of the unprocessed DCI and the DCI after LSM as a function of temperature under an argon atmosphere. Table 3 summarizes the cooling rate and temperature gradient at the surface of the molten pool along the SMB centerline for the investigated processing conditions.

The data implied that the cooling rate at the trailing edge of the molten pool decreased when the heat input level increased. Furthermore, an increase in the heat input significantly decreased the cooling rate behind the molten pool. From 1090 to 800 °C, the cooling rate of the region of the solidified melt-bead material behind the liquid/solid interface was about 360 and 130 °C/s for heat input levels of 600 and 1200 J/mm, respectively (note that the solidus temperature of the investigated DCI was estimated from the Scheil calculation to be approx. 1090 °C; Section 3.2). The temperature gradient in the trailing edge of a molten pool directly depends on the molten pool’s geometry, which in turn is significantly affected by the traverse speed [18,19]. In the case of the investigated DCI, for a given heat input level, the change in the traverse speed also significantly affected the cooling rate at the trailing edge of the molten pool. At a heat input of 600 J/mm (A1 and A2 processing conditions; Table 2), increasing the traverse speed notably decreased both the temperature gradient and the cooling rate at the trailing edge of the molten pool. For the A1 and A2 processing conditions, the cooling rates at the trailing edge of the molten pool were 495 ± 38 and 611 ± 28 °C/s, respectively (calculated from 1200–1090 °C). This was mainly attributed to the difference in the molten pool shape. Due to the higher traverse speed (3.33 mm/s), the A1 processing condition led to a significantly elongated molten pool. As a result, the distance over which the temperature decreased from its maximum value to the solidus temperature of the DCI substrate was significantly larger than in the A2 processing condition (traverse speed of 1.66 mm/s). On the other hand, it is reasonable to suggest that the large difference in the laser power between the A1 and A2 processing conditions (2000 and 1000 W, respectively) may have also affected the amount of energy that was absorbed by the molten pool. This suggestion is supported by the fact that the cross-sectional area of the bead that was produced at a laser power of 2000 W was about 40% larger than that of the bead that was produced at 1000 W. An increased absorption of laser light with an increase in laser intensity, at a constant traverse speed, was reported by Trapp et al. [20]. The cooling rate values of the region of the solidified melt-bead in the temperature range of 1090–800 °C did not significantly vary with changes in the traverse speed at a heat input of 600 J/mm.

### 3.2. Thermodynamic Calculations 

**Figure 5:**
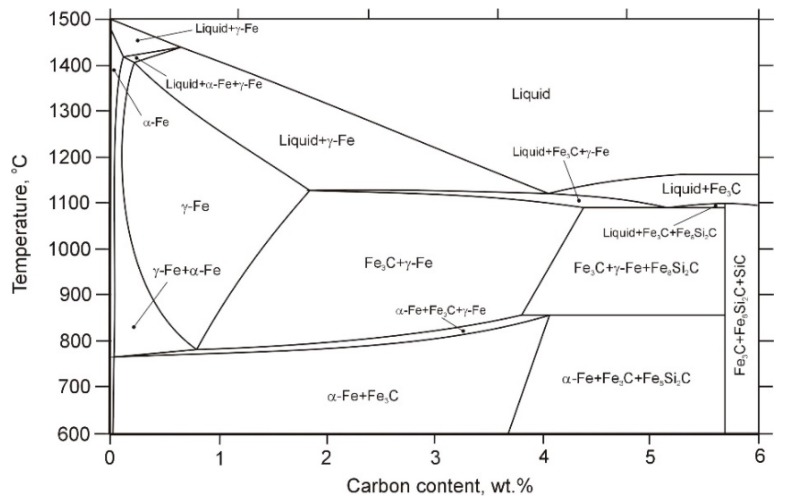
Isopleth at 2.5 wt% Si in the metastable equilibrium Fe-C-Si phase diagram.

**Figure 6:**
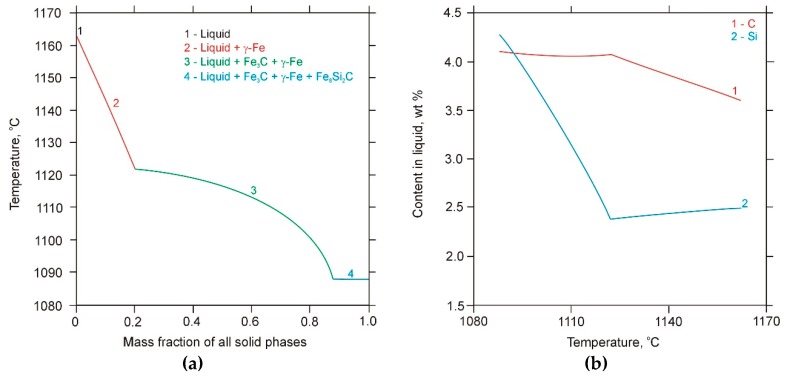
(**a**) Scheil solidification path for the Fe-3.6 wt% C-2.5 wt% Si alloy system; (**b**) the corresponding composition of the liquid as the function of temperature.

The metastable equilibrium phase diagram (i.e., considering the formation of the cementite phase instead of graphite) and the Scheil solidification path resulting from the thermodynamic calculations of a ternary Fe-3.6 wt% C-2.5 wt% Si alloy system are presented in Figure 5 and Figure 6a, respectively. The calculation showed that the first phase to precipitate in the molten pool was an austenite phase (γ-Fe). Further cooling indicated that a binary eutectic reaction occurred, and this led to the formation of ledeburite eutectic (γ-Fe+Fe_3_C). In contrast to the metastable equilibrium diagram, the Scheil model predicted that the solidification path ended with the formation of ternary eutectic γ-Fe/Fe_3_C/Fe_8_Si_2_C. Generally, the Scheil simulation results of the Fe-C-Si alloy system suggested the following solidification sequence in the DCI substrate: 

(1) Nucleation of primary austenite (~1165 °C):

Liquid → Liquid + γ-Fe.

(2) Binary eutectic reaction (~1120 °C ):

Liquid → Liquid + γ-Fe + Fe_3_C.

(3) Ternary eutectic reaction (~1090 °C ):

Liquid → Liquid + γ-Fe + Fe_3_C + Fe_8_Si_2_C.

According to the literature [21,22], the formation of the ternary eutectic γ-Fe/Fe_3_C/Fe_8_Si_2_C in an Fe-C-Si alloy system occurs at Si contents higher than 3.0 wt %. This is in good agreement with the Scheil calculation results for segregated alloying elements during the solidification of the investigated DCI (Figure 6b), which indicated that the C and Si contents in the liquid at the final solidification stage reached 4.1 and 4.3 wt %, respectively. The presence of eutectic γ-Fe/Fe_3_C/Fe_8_Si_2_C in the microstructure of the SMBs was confirmed by the microstructural investigations that are presented in Section 3.3.

### 3.3. Microstructural Analysis 

**Figure 7:**
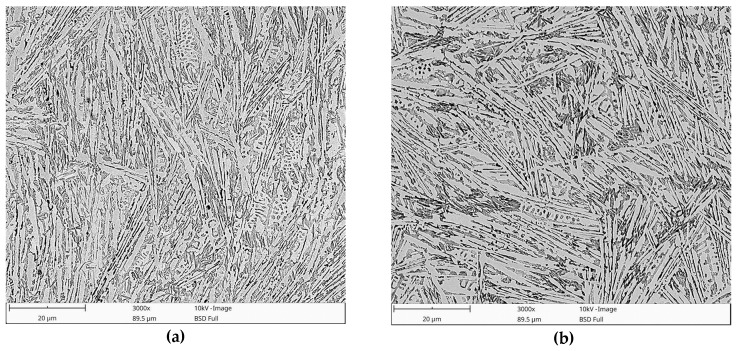

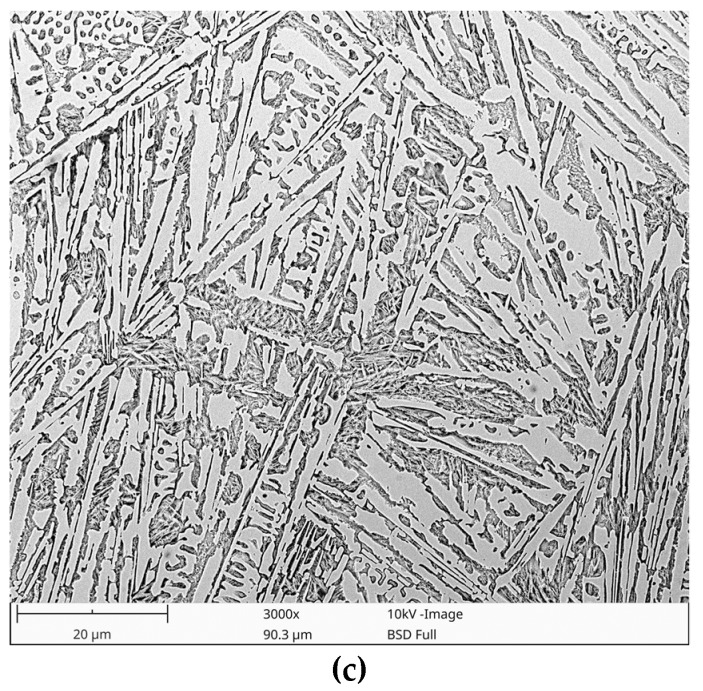
Backscattered electron (BSE) SEM micrographs that were taken of the undersurface area of (**a**) SMB1, (**b**) SMB2, and (**c**) SMB3 (Table 4).

**Figure 8:**
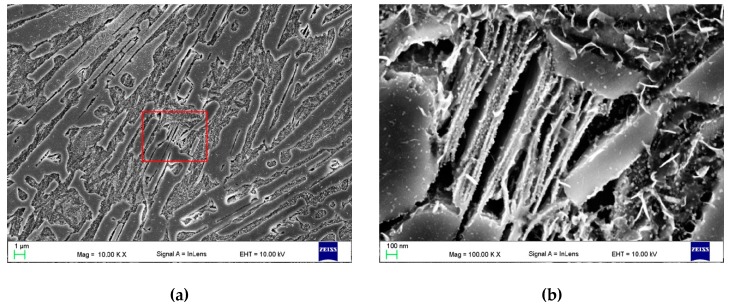

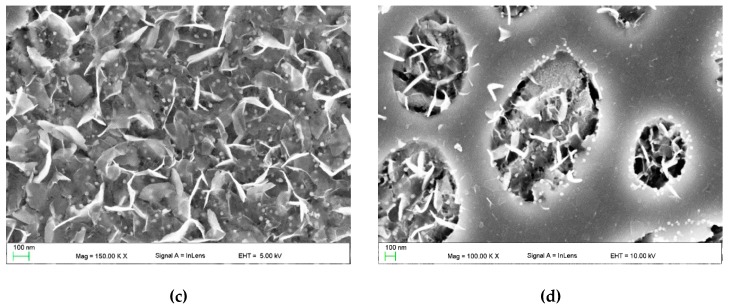
In-lens SEM images taken of SMB3 (~150 µm beneath the bead surface) showing (**a**) the distribution of ternary eutectic α-Fe/Fe_3_C/Fe_8_Si_2_C; (**b**) a detail from (a) showing the morphology of the α-Fe/Fe_3_C/Fe_8_Si_2_C eutectic (marked area); (**c**,**d**) a distribution of plate-like and spherical cementite precipitates in (c) the primary austenite grain and (d) eutectic austenite regions (Table 4).

Figure 7 shows BSE SEM images that were taken of the undersurface area of SMBs that were produced under the processing conditions listed in Table 2. The corresponding microstructural parameters of SMBs, based on a quantitative analysis of micrographs and XRD patterns, are presented in Table 4. In general, the microstructure of the melted zone contained primary austenite (γ_p_) dendrites transformed to martensite (partially or fully) and a ledeburite eutectic in the interdendritic regions that, in turn, was composed of a eutectic austenite (γ_e_) phase and a eutectic cementite phase. Additionally, the formation of fine eutectic-like structures was observed in the interdendritic regions, as shown in Figure 8a,b. Based on the results of thermodynamic calculations (Section 3.2) that predicted a ternary eutectic reaction at the final solidification stage under non-equilibrium conditions, it is reasonable to assume that the above fine eutectic-like structures were ternary eutectic α-Fe/Fe_3_C/Fe_8_Si_2_C. This assumption is also supported by the fact that the morphology of the observed ternary eutectic is consistent with that previously reported for the Fe-C-Si alloy system [23]. The above results indicate a good agreement between the experimental results and those that were calculated under the Scheil condition.

Due to the high carbon content in the processed DCI (3.6 wt%), the eutectic regions (ledeburite eutectic structure) formed a continuous network surrounding the γ_p_ dendrites. The investigated solidification conditions did not provide a substantial change in the fraction of the ledeburite eutectic. The fraction of the eutectic cementite was approx. 53 vol%. In contrast, the cooling rate altered the morphology of the γ_p_ dendrites and ledeburite eutectic. Generally, as the cooling rate decreased, the γ_p_ dendrites and ledeburite eutectic became coarser (Figure 7). However, the difference in the solidification conditions between the two analyzed cases at a heat input of 600 J/mm (SMB1 and SMB2, Table 3 and Table 4) had no marked influence on the dimensions of the solidification structure.

The metallographic data presented in Table 4 indicate a strong correlation between the fraction of the retained austenite (γ_r_) and the cooling rate. Generally, an increase in the cooling rate increases the γ_r_ fraction. At a given heat input, the γ_r_ fraction is affected by the traverse speed. When comparing the microstructural data of SMB1 and SMB2, a larger fraction of γ_r_ existed in the SMB2 microstructure. When considering that the cooling rate behind the molten pool was essentially the same in both cases, this difference can be attributed to the different cooling rates during solidification (at the trailing edge of the molten pool). In both the casting process and laser surface treatment of cast irons, it has been reported that under non-equilibrium cooling conditions, the austenite becomes supersaturated with carbon and suppresses the martensitic transformation [24,25,26]. Thus, it seems very reasonable to assume that the faster cooling rates during solidification increased the carbon content in the austenite phase and inhibited the martensitic transformation. This assumption is supported by the values of the γ_r_ austenite lattice parameter, which were estimated to be 0.3597 and 0.3611 nm in SMB1 and SMB2, respectively. This confirms an increase in the carbon content in the γ_r_ austenite as the cooling rate increased during solidification. Considering the microstructural parameters of SMB3, it is reasonable to conclude that the cooling rates during the fabrication of this melted zone ensured a nearly complete martensitic transformation of the γ_p_ and γ_e_ austenite phases.

**Figure 9:**
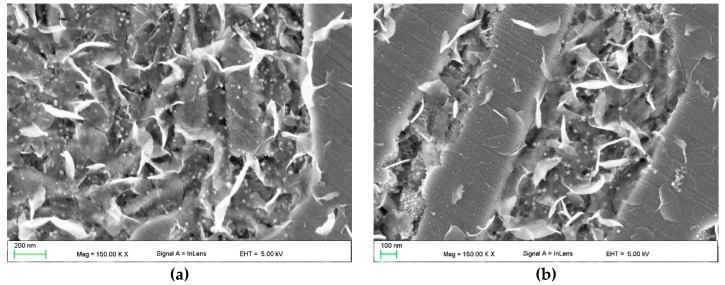
In-lens SEM images showing the distribution of plate-like and spherical cementite precipitates throughout the undersurface area of SMB1 (~150 µm beneath the bead surface): (**a**) primary austenite grain; (**b**) ledeburite eutectic region.

**Figure 10:**
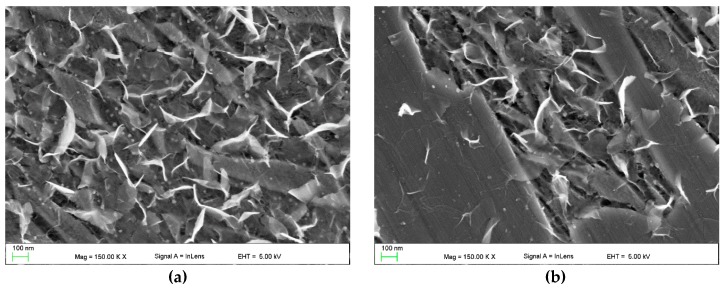
In-lens SEM images showing the distribution of plate-like and spherical cementite precipitates throughout the undersurface area of SMB2 (~150 µm beneath the bead surface): (**a**) primary austenite grain; (**b**) ledeburite eutectic region.

**Figure 11:**
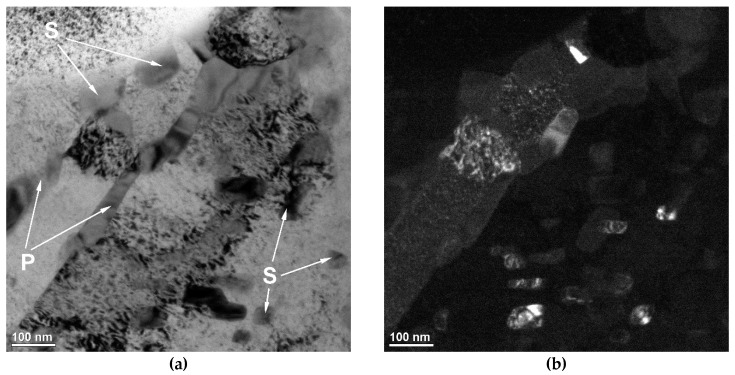
(**a**) Bright field and (**b**) dark-field TEM images showing the nanometric plate-like (P) and spherical (S) cementite precipitates in the primary austenite grains in SMB3 (Table 4).

Detailed SEM investigations (Figure 8, Figure 9 and Figure 10) revealed the presence of bright nanometric precipitates that exhibited plate-like and spherical morphologies in the γ_p_ and γ_e_ regions. Figure 11 shows the TEM micrographs of the γ_p_ region (grain) in SMB3. The plate-like and spherical precipitates were identified as the cementite phase by SAED. As shown in Figure 8, Figure 9 and Figure 10, in the entire range of investigated solidification conditions, a continuous network of plate-like cementite precipitates was formed in both the γ_p_ and γ_e_ regions. Spherical cementite precipitates were also observed in all SMBs; however, SMB2—the bead that was produced at the highest cooling rate—showed the lowest fraction of spherical cementite in both the γ_p_ and γ_e_ regions. This indicates that increasing the cooling rate inhibited the precipitation of spherical cementite. According to a TEM micrograph (Figure 11), plate-like cementite precipitates were located at the martensite lath boundaries, whereas spherical precipitates seemed to be located at both the martensite lath boundaries and inside the martensite laths. Note that the γ_r_ austenite fraction in SMB3 was negligible. Based on these observations, it is reasonable to assume that the nanometric plate-like and spherical precipitates were secondary and tertiary cementite, respectively. 

### 3.4. Hardness Analysis 

**Figure 12:**
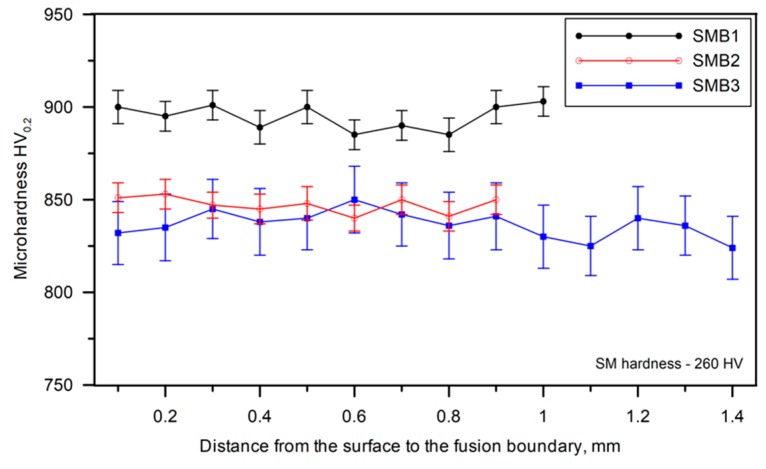
Hardness profiles of SMBs (Table 4).

The hardness profiles of the SMBs are compared in Figure 12. It is interesting that, despite the considerable differences in the solidification conditions, the overall hardness profiles of the SMBs were quite similar. Due to the high fraction of the eutectic cementite, the hardness of the melted zone was influenced by both the γ_r_ fraction and the morphology of the ledeburite eutectic. The formation of very fine ledeburite due to the high cooling rate during solidification elevated the overall hardness of the melted zone. The effect of the ledeburite eutectic morphology on the hardness of the melted zone can be observed when comparing hardness profiles of SMB1 and SMB3. Despite its significantly higher γ_r_ fraction, SMB1 showed a higher average hardness (895 HV0.2) than SMB3 (837 HV0.2). This difference can be directly attributed to a difference in the morphology of the ledeburite eutectic. In turn, comparing the hardness data of SMB1 and SMB2, i.e., beads with a similar ledeburite eutectic morphology, shows that increasing the γ_r_ fraction decreased the overall hardness of the melted zone.

## 4. Conclusions

The influence of solidification conditions on the microstructure and hardness of laser-surface-melted DCI was investigated. The thermal conditions in the molten pool were examined with IR thermography. The results showed that increasing the cooling rate during solidification increased the carbon content in the austenite phase, which inhibited the martensitic transformation. Additionally, increasing the cooling rate during solidification refined the ledeburite eutectic. The hardness of the melted zone was affected by the fraction of the retained austenite, as well as the morphology of ledeburite eutectic. Due to the high fraction of ledeburite, its morphology had a significant influence on the hardness of the melted zone. Under the investigated processing conditions, the solidification of the melted zone terminated with the Liquid → γ-Fe + Fe_3_C + Fe_8_Si_2_C reaction. Furthermore, it was revealed that the primary and eutectic austenite regions contained a network of plate-like secondary cementite precipitates and nanometric spherical tertiary cementite precipitates. Increasing the cooling rate inhibited the precipitation of the spherical tertiary cementite.

## Figures and Tables

**Table 1:** Chemical composition of the used DCI grade EN-GJS-700-2 (wt %).

C	Si	Cu	Mn	Cr	Ni	S	P	Fe
3.60	2.51	0.78	0.25	0.02	0.04	0.008	0.016	balance

**Table 2:** Selected processing conditions of laser surface melting (LSM).

Processing Conditions No.	Laser Power (W)	Traverse Speed (mm/s)	Heat Input ^1^ (J/mm)
A1	2000	3.33	600
A2	1000	1.67	600
A3	1500	1.25	1200

^1^ defined by the ratio of the laser power and the traverse speed.

**Table 3:** Values of the cooling rate *(GR)* and temperature gradient (*G*) at the surface of the molten pool along the SMB centerline for different processing conditions.

Processing Conditions No. (Table 2)	Temperature Range (°C)			
	1200–1090 ^2^		1090–800	
	*GR*^1^ (°C/s)	*G*^1^ (°C/mm)	*GR*^1^ (°C/s)	*G*^1^ (°C/mm)
A1	495 ± 38	149 ± 12	326 ± 32	98 ± 10
A2	611 ± 28	366 ± 17	337 ± 18	202 ± 10
A3	97 ± 22	78 ± 17	127 ± 11	102 ± 9

^1^ Mean value and the standard deviation of three measurements; ^2^ the solidus temperature of Fe-3.6 wt% C-2.5 wt% Si alloy system that was determined by using the Scheil calculation.

**Table 4:** Microstructural parameters of SMBs.

Bead Designation	Processing Conditions No. (Table 2)	α-Fe (Martensite) Fraction (wt %)	Retained Austenite Fraction (wt %)	Eutectic Cementite Fraction (vol %)
SMB1	A1	25 ± 3	22 ± 3	53 ± 4
SMB2	A2	16 ± 3	30 ± 3	52 ± 4
SMB3	A3	44 ± 4	3 ± 1	53 ± 5
